# Supplementary figures and images for: Exploring heterogeneity in treatment effects: The impact and interaction of asset-based wealth and mass azithromycin distribution on child mortality
Source: PLoS One. 2026 Feb 9;21(2):e0341665. doi: 10.1371/journal.pone.0341665 (PMC12885275; doi:10.1371/journal.pone.0341665)

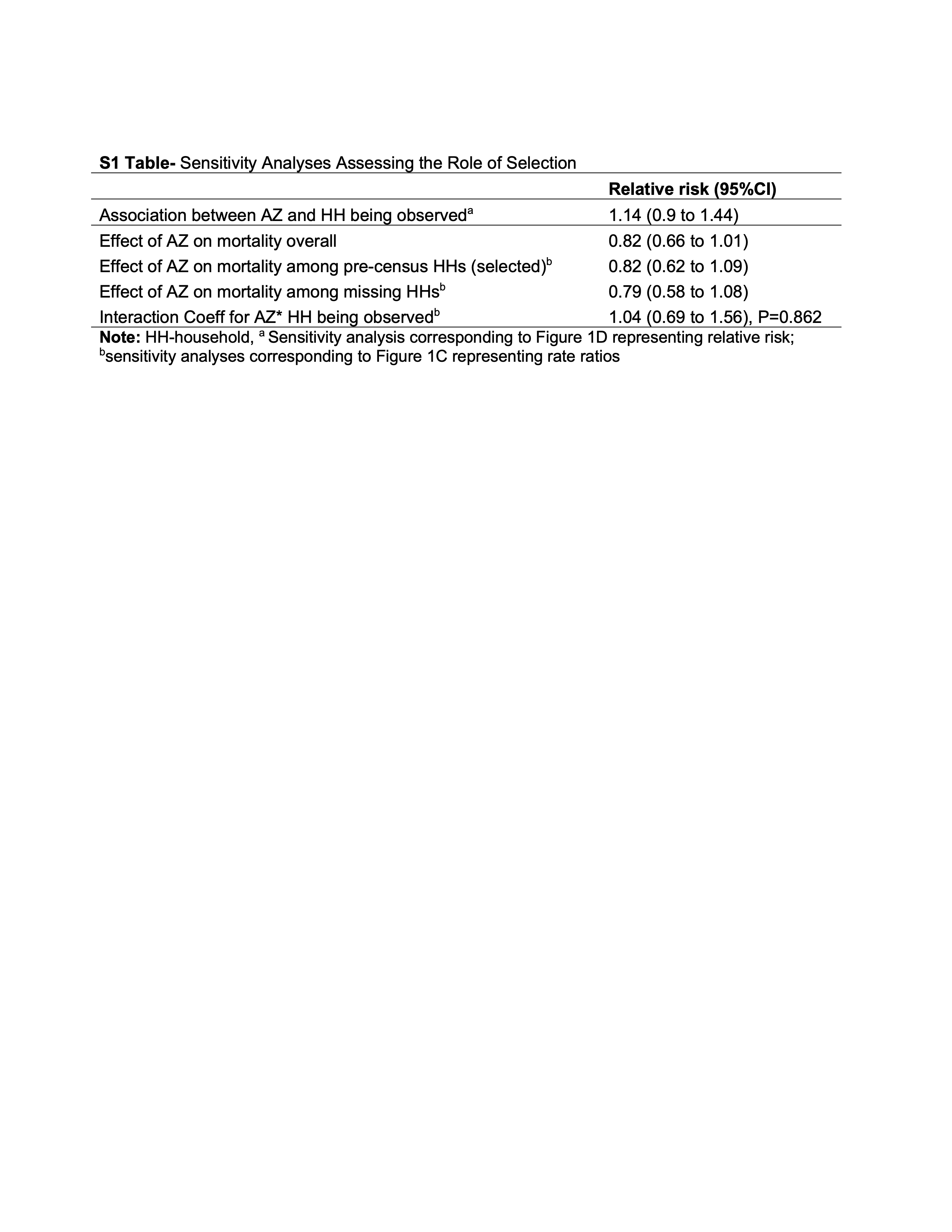

Supplement: S1 Table — (TIFF) [file pone.0341665.s001.tiff]

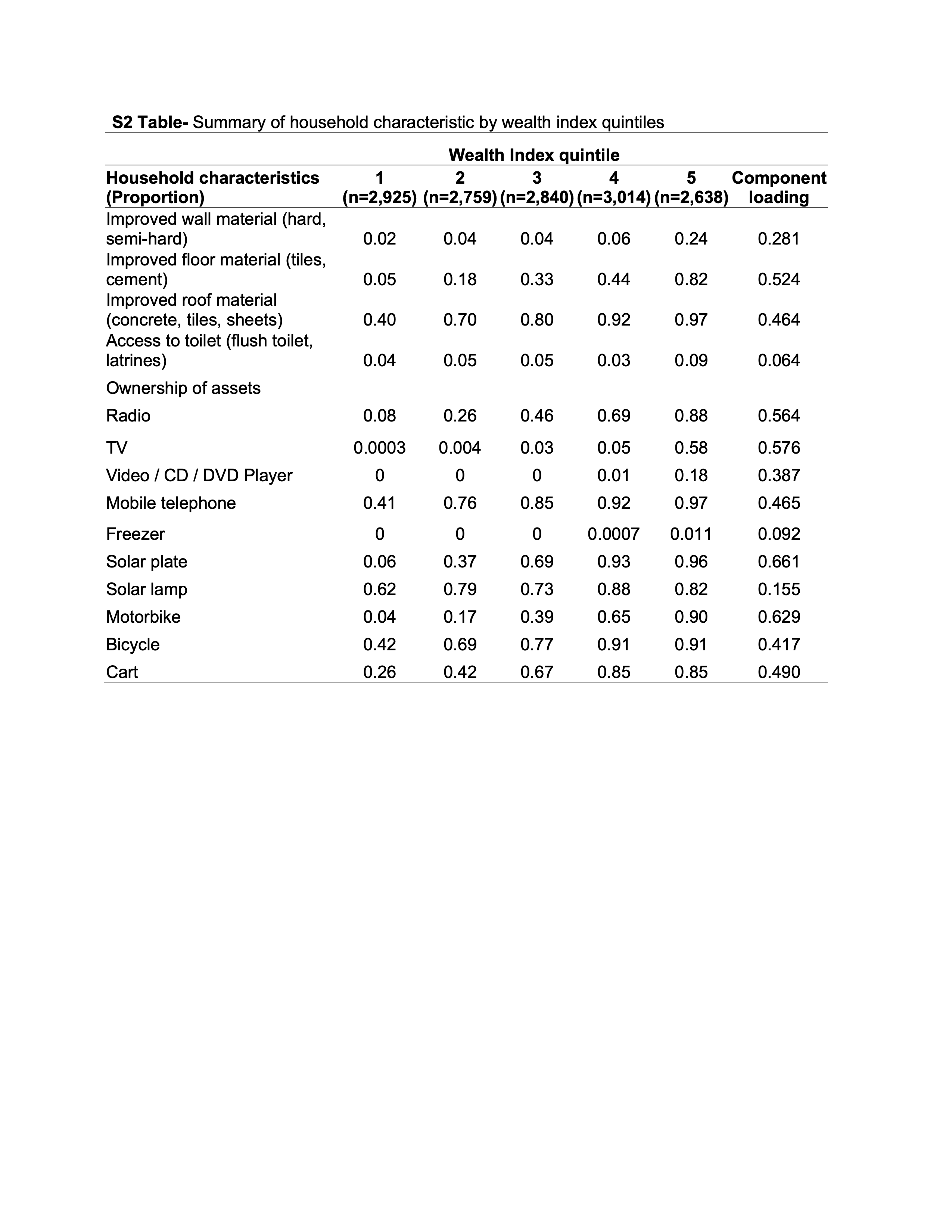

Supplement: S2 Table — (TIFF) [file pone.0341665.s002.tiff]

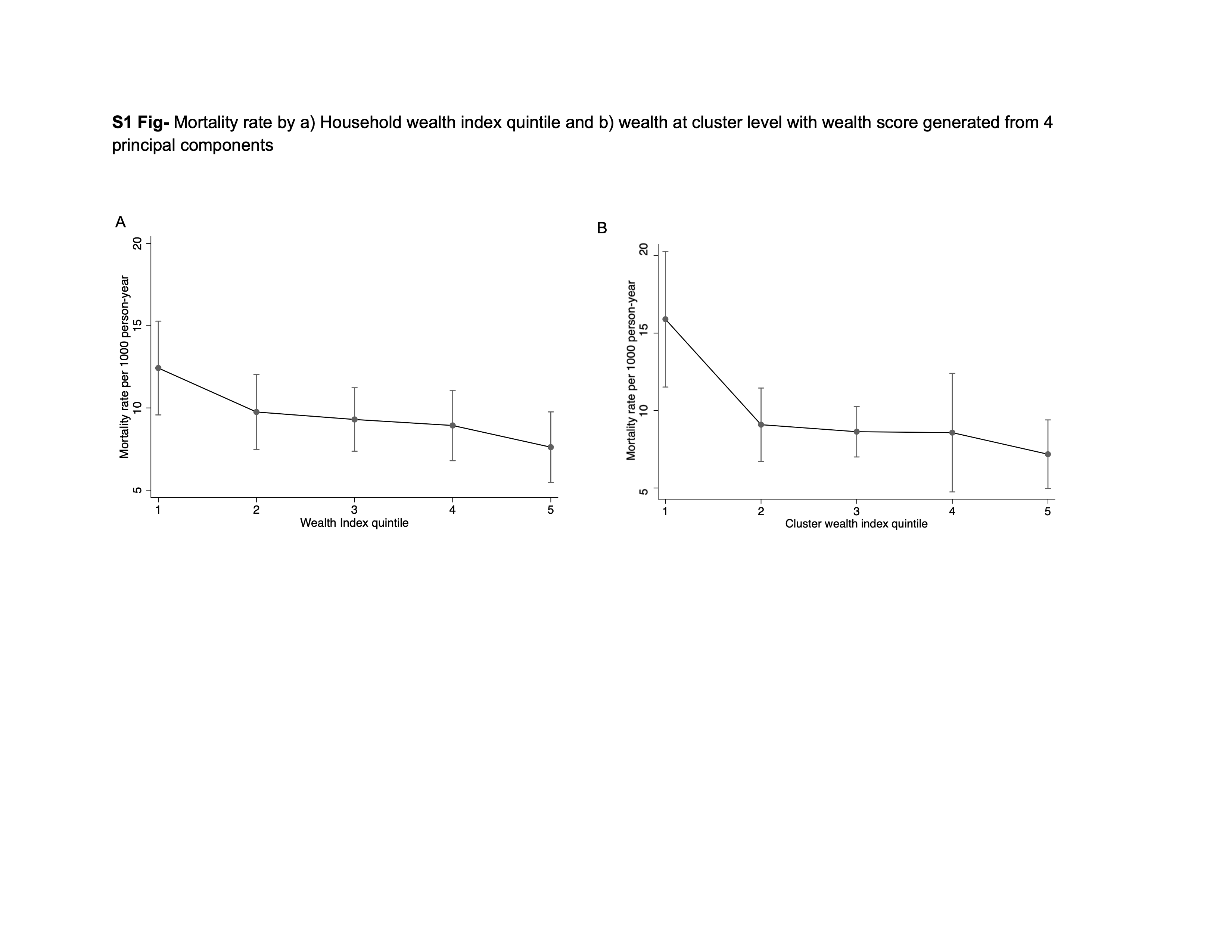

Supplement: S1 Fig — (TIFF) [file pone.0341665.s003.tiff]

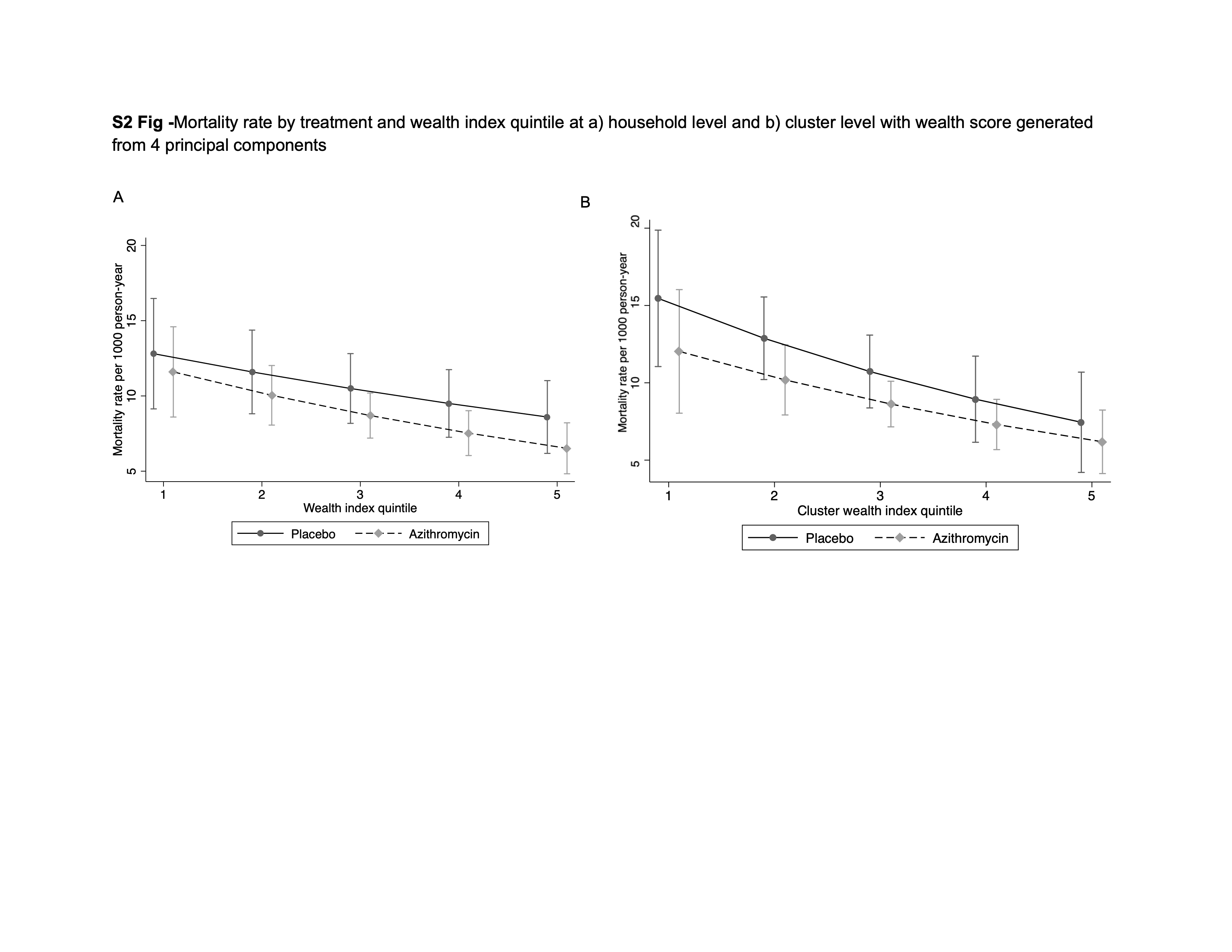

Supplement: S2 Fig — (TIFF) [file pone.0341665.s004.tiff]
