# Supplementary material for: Exploring heterogeneity in treatment effects: The impact and interaction of asset-based wealth and mass azithromycin distribution on child mortality
Source: PLoS One. 2026 Feb 9;21(2):e0341665. doi: 10.1371/journal.pone.0341665 (PMC12885275; doi:10.1371/journal.pone.0341665)
Supplement: Attachment — Submitted filename: Response to reviewers_PLOS One_Aug23.pdf [file pone.0341665.s007.pdf]

August 23, 2025

Academic Editor: Saidul Abrar, MBBS, MPH, Ph.D

PLOS One

RE: MANUSCRIPT ENTITLED, "Exploring Heterogeneity in Treatment Effects: The Impact and Interaction of Asset-Based Wealth and Mass Azithromycin Distribution on Child Mortality" PONE-D-25-23672

Dear Dr. Saidul Abrar,

We are grateful to have the opportunity to respond to the Reviewers' and Editor's comments and queries. We truly appreciate their time and effort in assessing our manuscript. Our responses follow below:

Journal Requirements:

1. When submitting your revision, we need you to address these additional requirements.

Please ensure that your manuscript meets PLOS ONE's style requirements, including those for file naming. The PLOS ONE style templates can be found at https://journals.plos.org/plosone/s/file?id=wjVg/PLOSOne_formatting_sample_main_body.pdf and https://journals.plos.org/plosone/s/file?id=ba62/PLOSOne_formatting_sample_title_authors_affiliations.pdf

Response: We have revised the manuscript to align with PLOS ONE's formatting requirements, using the style templates provided.

2. Thank you for stating in your Funding Statement:
The CHAT trial was supported by the Gates Foundation (grant number OPP1187628). Research reported in this manuscript was also supported by the National Institutes of Health Eunice Kennedy Shriver National Institute of Child Health & Human Development (NIH/NICHD) F31 Award (1F31HD114434-01A1: E.A.G.).

Please provide an amended statement that declares *all* the funding or sources of support (whether external or internal to your organization) received during this study, as detailed online in our guide for authors at http://journals.plos.org/plosone/s/submit-

now. Please also include the statement “There was no additional external funding received for this study.” in your updated Funding Statement.
Please include your amended Funding Statement within your cover letter. We will change the online submission form on your behalf.

Response: As suggested, we have added in the funding statement that no additional external funding was received for this study.

3. In the online submission form, you indicated that your data will be submitted to a repository upon acceptance. We strongly recommend all authors deposit their data before acceptance, as the process can be lengthy and hold up publication timelines. Please note that, though access restrictions are acceptable now, your entire minimal dataset will need to be made freely accessible if your manuscript is accepted for publication. This policy applies to all data except where public deposition would breach compliance with the protocol approved by your research ethics board. If you are unable to adhere to our open data policy, please kindly revise your statement to explain your reasoning and we will seek the editor's input on an exemption.

Response: Data will be made available in OSF upon acceptance. We are currently finalizing the process to publish the data. We have noted this in the manuscript.

4. Your ethics statement should only appear in the Methods section of your manuscript. If your ethics statement is written in any section besides the Methods, please move it to the Methods section and delete it from any other section. Please ensure that your ethics statement is included in your manuscript, as the ethics statement entered into the online submission form will not be published alongside your manuscript.

Response: We have ensured the ethics statement appears exclusively in the Methods section as required.

5. If the reviewer comments include a recommendation to cite specific previously published works, please review and evaluate these publications to determine whether they are relevant and should be cited. There is no requirement to cite these works unless the editor has indicated otherwise.

Additional Editor Comments:

Kindly revise your manuscript as per the comments of reviewers. Thanks

Reviewers' comments:

Reviewer's Responses to Questions

Comments to the Author

Reviewer #1: As the statistical reviewer I will focus on methods and reporting.

Major
1) One major concern is where AZ exposure sits on the causal pathway in relation to SES and the primary outcome. I appreciate this was on the back of an RCT, so in principle everyone should be exposed. but can the authors explore and confirm that? Perhaps another short section on wealth status and AZ exposure in the methods section. In other words, the study's observational secondary analysis design limits causal inference regarding the interaction between azithromycin and wealth, particularly in the absence of randomisation by wealth strata.

Response: We appreciate the reviewer's point about the limitations introduced by the lack of randomization of wealth. AZ was randomized at the community level, which implies that, by design, treatment assignment is independent of household or community wealth status, demographics, or other factors. This eliminates any influence on AZ exposure. Furthermore, treatment coverage (of assigned treatment, AZ or placebo) was approximately 90% in both arms, and all communities received their assigned treatment per protocol, ensuring no differential implementation across wealth strata. As suggested, to confirm the balance of treatment assignment across SES strata, we evaluated the proportion treated by wealth quintile. AZ and placebo were distributed approximately equally across all five wealth quintiles, with no systematic differences (e.g., 58.8% AZ in the poorest quintile vs. 50.0% in the wealthiest quintile). These percentages for each wealth quintile have been added to the Results section to clarify this point.

While we acknowledge the lack of randomization of wealth may limit causal interpretation of the interaction, our analysis helps us evaluate whether the causal effect of AZ estimated through randomization, varies across levels of wealth. This approach aligns with standard effect modification analyses in RCTs and provides policy-relevant insight into whether AZ offers differential benefits by socioeconomic status. We have also avoided language suggesting causal interaction, in line with this distinction.

We have added the following in the beginning of the results section. "The overall coverage of MDA was approximately 90% for both AZ and placebo arms. The proportion of communities that received AZ in each wealth quintile was: 58.8% (quintile 1, poorest), 47.1% (quintile 2), 58.8% (quintile 3), 50.0% (quintile 4), and 50.0% (quintile 5, wealthiest)."

2) why isn't the village cluster included in the models as an additional higher level random intercept? a 3 level mixed effects model would be preferable to the current model (clarify which command in Stata you used and you will use btw) with robust

standard errors.

Response: We appreciate the suggestion regarding model choice. For the household-level interaction analysis, we used a Poisson regression model (*poisson in Stata)* with a log link, person-years as an offset, and robust standard errors clustered at the level of randomization (*vce(cluster clusterid))* to account for correlation between households within the same cluster. Clusters and villages were used interchangeably throughout the manuscript. We have now updated it to clusters or communities. Our intent was to estimate how the effect of azithromycin varies by wealth at the population level (i.e., marginal effects), rather than to model variation between communities, as would be done in a mixed-effects framework.

We agree, however, that it is important to assess whether the results are sensitive to the choice of model. In response to this comment, we conducted a sensitivity analysis using a mixed-effects Poisson model *(mepoisson)* with a random intercept for cluster.

The results from this model were consistent with our main analysis. Specifically, the interaction between azithromycin and wealth remained non-significant on both the multiplicative and additive scales: Multiplicative interaction coefficient: 0.97 (95% CI: 0.86 to 1.09, p = 0.632); Additive interaction (RERI): –0.03 (95% CI: –0.19 to 0.13, p = 0.724)

This additional analysis and its findings are now reported in the corresponding Methods and Results sections and noted in a footnote to Table 4.

3) Power will be pretty low for the interaction analysis, something the authors state in the limitations section, but they discuss statistical power in the abstract and the results. the abstract and the discussion needs to reflect that power probably too low to pick up anything but a very large association. so be more conservative when you discuss the findings from the interaction analysis.

Response: We agree and have revised the abstract and discussion to more explicitly acknowledge the limited power for detecting interaction effects. We now note that the analyses may have been underpowered to detect small to moderate effects. We have added the phrase: “While the analysis may have been underpowered to detect modest interaction effects, …” in both sections.

4) The strategy for dealing with missing data needs to be clearly stated. There is a brief mention in the context of the sensitivity analysis, but as per the STROBE statement

(which you should follow) a clear explanation is needed about the approach (in this case listwise deletion). My issue is why multiple imputation was not conducted as a sensitivity analysis (or the main analysis preferably). This is a major concern since nearly half the households from the household-level analysis are excluded due to missing pre-census data, something that could introduce selection bias, even if sensitivity analyses suggest minimal impact.

Response: As suggested, we have clarified in the Methods section that households with missing pre-census data were excluded from the household-level analysis using listwise deletion (i.e., complete case analysis) to explicitly state this approach and align with the STROBE guidelines on missing data reporting.

Multiple imputation was considered but was not feasible in this case. The pre-census data had nearly all household-level variables, which were missing for these excluded households, leaving only mortality, treatment assignment (AZ versus placebo), follow-up time, and community ID documented in the main trial. These variables were insufficient to reliably impute missing wealth scores.

In addition to conducting parallel analyses at the community level, we assessed whether the missingness process may have introduced selection bias. Sensitivity analyses suggested that missingness was not associated with study arm and that the effect of AZ on mortality was similar among included and excluded households. This provides some reassurance that the exclusion due to missing data did not substantially bias the results. We have also acknowledged this issue and our sensitivity findings in the Limitations section.

6) PCI may oversimplify the multidimensional nature of socioeconomic status and introduce measurement error, especially given the relatively low variance explained by the first component (discuss as a limitation).

Response: As suggested, we have highlighted the potential oversimplification of the multidimensional nature of wealth. The limitations paragraph has been revised to explicitly acknowledge that the PCA may not fully capture the complexity of household socioeconomic status and could introduce measurement error.

> "Second, there is a potential for misclassification or measurement error, as the asset-based wealth index may not fully capture the complexity *and multidimensional nature* of wealth within households. Although this limitation could affect the accuracy of our analyses, it would likely impact all communities similarly and would not be differential *measurement error* by mortality levels. The extent to which these scores adequately represent wealth can also be affected by the PCA. Although the proportion of variability explained by the first component was not large and *may therefore be an*

oversimplification, the combination of the first 4 components, which explained a much larger proportion of variability, generated similar findings."

7) The use of community-level averages to impute wealth in cluster-level analyses assumes homogeneity within clusters, potentially masking intra-cluster variability.

Response: As suggested, we have acknowledged this limitation and added the following statement: "This approach assumes homogeneity within clusters, potentially masking intra-cluster variability in wealth."

Minor

1) clarify for readers that the random components are ignored with the margins command.

Response: Thank you for this comment. We used the margins command only with the Poisson regression model that included clustered robust standard errors but no random effects. Therefore, no random components were ignored in the estimation. We have clarified in the Methods section that these estimates reflect marginal effects. For the mixed-effects Poisson model used in the sensitivity analysis, we directly reported the interaction coefficients on both the multiplicative and additive scales.

2) rephrase "robust standard errors clustered at the cluster level to account for the cluster-level treatment in..."

Response: As suggested, we have rephrased the text to: "robust standard errors clustered at the level of randomization (i.e., community/cluster) to account for cluster-level treatment in the household-level analysis."

3) Another limitation to discuss is potential unmeasured confounders such as nutritional status, immunization coverage, or environmental exposures which may influence both wealth and mortality

Response: Thank you for the suggestion. We have updated the limitations section to acknowledge additional potential unmeasured confounders. The revised text now reads:

"Lastly, since we did not have additional data on clusters and households, we could not adjust for other potential confounders of the wealth–mortality association, such as bed net access, nutritional status, immunization coverage, or environmental exposures."

Reviewer #2: The authors have done tremendous work for the clinical trials, but still the manuscript needs to be revised for some of the suggestions prior to being published.

1) The abstract section must be revised, as a lot of changes are recommended in the file section.

Response: As suggested, we have revised the abstract for clarity, scientific tone, and grammar, and incorporated changes in the sections highlighted in the file.

2) The manuscript must be revised for typographic mistakes and grammatical errors as highlighted in the file section.

Response: Thank you for your comment. We have reviewed the manuscript for typographic and grammatical errors and revised the text for clarity, grammar, and flow throughout, including in the highlighted sections.

3) The cluster clinical trials are not explained. Are the trials single-blind or double-blind?

Response: We have added in the Methods section, under Data Collection, that the CHAT trial was double-masked and placebo-controlled.

4) The result and discussion portion should be updated with strong, updated references.

Response: As suggested, we have replaced older citations, such as Peters (2008) and Liu (2012), with more recent and relevant publications. We have also reviewed all references to ensure they are up-to-date and appropriately support the results and discussion.

5) The conclusion section should be written scientifically with sophisticated words.

Response: We have revised the conclusion using a more scientific and polished tone, while maintaining the clarity and key messages of the original text.

6) There is no "future prospect" section included in the manuscript?

Response: We have made the implications for future research and programmatic focus, which are outlined in the concluding paragraph, more explicit. Specifically, the study highlights that despite reductions in overall child mortality, significant wealth-related disparities persist. We found no evidence to support prioritization of AZ distribution based on wealth status. Therefore, our findings support continued broad-based mass drug administration programs while also underscoring the need for further research into

strategies that address the structural and contextual drivers of differential mortality across socioeconomic strata.

7) If the data showed in the manuscript is directly taken from the patients, then the manuscript is acceptable for publication, but if the analyzed data is taken from any already collected source, then the manuscript should not be published.

Response: Thank you for your comment. All data used in this analysis were collected directly from participants as part of the CHAT trial and the accompanying pre-census survey. The census data were collected prospectively by the study team every 6 months, and the pre-census wealth data were also gathered directly from households prior to trial implementation.

We respectfully note that analyses using previously collected data can also be scientifically valid and valuable when conducted with rigorous and appropriate methodology.

8) Which software was used to analyze the data?

Response: We have specified the following at the end of the Methods section: "SAS 9.4 (SAS Institute, Cary, NC) was used for data cleaning and for generating the datasets for analysis. Stata version 14.2 (StataCorp, College Station, TX) was used for all other analyses."

We wish to reiterate our gratitude for being given the opportunity to respond to the Reviewers' comments as well as for being considered for publication in PLOS One Journal. If there are any further questions or comments, please do not hesitate to contact me.

Sincerely,

Elisabeth Gebreegziabher, PhD, MPH
