# Supplementary material for: Exploring heterogeneity in treatment effects: The impact and interaction of asset-based wealth and mass azithromycin distribution on child mortality
Source: PLoS One. 2026 Feb 9;21(2):e0341665. doi: 10.1371/journal.pone.0341665 (PMC12885275; doi:10.1371/journal.pone.0341665)
Supplement: Attachment — Submitted filename: Response to reviewers_PLOS One_Dec12.docx [file pone.0341665.s008.docx]

December 12, 2025

Staff Editor: Alejandro Torrado Pacheco, PhD

PLOS One

**RE:**	**MANUSCRIPT ENTITLED, “Exploring Heterogeneity in Treatment Effects: The Impact and Interaction of Asset-Based Wealth and Mass Azithromycin Distribution on Child Mortality” PONE-D-25-23672**

Dear Dr. Alejandro Torrado Pacheco,

We are grateful to have the opportunity to respond to the Reviewers’ and Editor’s comments and queries. We truly appreciate their time and effort in assessing our manuscript. Our responses follow below:

 
**Review Comments to the Author**

Reviewer #1: I am satisfied with the authors' responses and the resulting changes to the paper. I have nothing else to add.

**Response**: Thank you! We appreciate the reviewer’s time and helpful feedback.

Reviewer #3: This is an interesting study examining the effect and interaction of asset-based wealth and mass azithromycin (AZ) distribution on child mortality.

1. On first reading, I found the indication for AZ unclear, even with my background in pharmacy. I had to refer to the CHAT trial to infer the context of this study. The authors should also describe the indication for AZ treatment in the CHAT trial (e.g., seasonal chemoprevention of malaria) within the manuscript for better context and accuracy.

**Response**: We have clarified the indication and rationale for azithromycin use in the CHAT trial to provide appropriate clinical context. We now note that azithromycin was distributed biannually to children as part of a community-based intervention aimed at reducing childhood mortality. Although azithromycin is primarily used to treat bacterial infections, its potential mortality benefit in mass drug administration programs has been attributed to its broad antimicrobial activity against respiratory and enteric pathogens common in low-resource settings.

We have added the following in the methods line 134:

“In the CHAT trial, azithromycin was distributed biannually to children as part of a community-based intervention aimed at reducing childhood mortality. Although azithromycin is an antibiotic primarily used to treat bacterial infections, its potential mortality benefit in mass drug administration programs has been attributed to its broad antimicrobial activity, including against respiratory and enteric pathogens common in low-resource settings.”

2. The authors used the mass distribution of AZ as an example of an intervention aimed at reducing child mortality, suggesting that AZ could buffer against disparities in mortality by addressing treatment gaps. However, the use of the term “AZ distribution for prevention of mortality” (line 120) may be too strong, as AZ does not have such an indication. A more accurate phrasing might be “prevention of mortality related to infection.”

**Response**: As suggested, we have replaced the phrase “for prevention of mortality” with “for prevention of infection-related mortality” throughout the manuscript (introduction, line 77 and 87, and Methods line 134). This more accurately reflects the intended mechanism.

3. The authors should justify why AZ, a macrolide antibiotic, was chosen when other antibiotics (e.g., broad-spectrum antibiotics) — potentially cheaper alternatives — could also be used to treat childhood infections. Furthermore, if AZ is being considered in relation to malaria, clarification is needed on why an antibiotic was selected over an antimalarial agent.

**Response**: We appreciate the reviewer’s comment. We have added justification for the use of azithromycin in the Introduction. Specifically, we clarify that azithromycin was selected in the CHAT trial based on strong prior evidence from large randomized controlled trials—most notably the MORDOR trial—which demonstrated reductions in all-cause childhood mortality following community-level mass distribution of azithromycin in sub-Saharan Africa. These findings motivated subsequent trials, including CHAT, to further evaluate its population-level impact. We also highlight azithromycin’s favorable pharmacologic and safety profile, including its long half-life, broad antimicrobial spectrum (covering respiratory and enteric pathogens), and demonstrated feasibility for use in mass drug administration programs. In our study setting, malaria prevention was addressed through routine seasonal malaria chemoprevention (SMC) programs, and azithromycin was not intended to replace antimalarial agents but rather to act as a complementary intervention targeting other major infectious causes of childhood mortality such as pneumonia and diarrhea.

We have added the following text in the Introduction (line 77):

AZ was selected for mass-distribution trials based on strong prior evidence from large randomized controlled trials, including the MORDOR study in Malawi, Niger, and Tanzania, which demonstrated significant reductions in all-cause childhood mortality following community-wide AZ administration.[8] These results led to further investigations, including the CHAT trial in Burkina Faso and the AVENIR trial in Niger, to evaluate whether these benefits could be replicated in other populations and contexts.[9, 10, 11] The favorable pharmacologic properties of AZ including its long half-life, broad antimicrobial activity against respiratory and enteric pathogens, and well-established safety profile, make it suitable for large-scale community interventions.[12]

4. Line 193: Please specify the variables included in the Poisson regression model. Additionally, provide justification for using the principal component analysis method instead of factor analysis.

**Response**: As suggested, we have clarified the model specification and the rationale for using principal component analysis (PCA). In the Statistical Analyses Methods section, line 215, we added:

“… Poisson regression model for the wealth - mortality analyses included child mortality as the outcome, the household wealth index as the main exposure, person-years at risk as an offset, and robust standard errors clustered at the community level to account for the cluster-level treatment.” As noted in the manuscript, distance to the nearest health facility, previously identified as a strong determinant of child mortality and correlated with wealth was included as an adjustment variable in the sensitivity and adjusted models.

For the wealth-AZ interaction models, we have clarified (line 249) that we used Poisson regression models similar to those described above, including both the main effects of wealth and AZ, as well as their interaction term to assess interaction on a multiplicative scale.

We also expanded the description of the PCA approach (line 194) as follows: “PCA was used to generate the wealth index because it efficiently summarizes correlated asset variables into a single composite score and is widely used for constructing asset-based wealth indices in demographic and health studies, including those conducted in sub-Saharan Africa.”

This approach captures the largest proportion of variability in socioeconomic status and maintains comparability with DHS and other population-based surveys. Factor analysis, while conceptually similar, assumes underlying latent constructs and requires additional distributional assumptions that were not necessary for the purpose of deriving an empirical wealth index.

5. Minor:
   Results — Table 3: Emphasize or clearly mark the statistically significant values.

**Response**: Thank you for the suggestion. All estimates presented in Table 3 were statistically significant at α = 0.05 (P < 0.05). We have added a note below the table to clarify this convention.

We wish to reiterate our gratitude for being given the opportunity to respond to the Reviewers’ comments as well as for being considered for publication in PLOS One Journal. If there are any further questions or comments, please do not hesitate to contact me.

Sincerely,

Elisabeth Gebreegziabher, PhD, MPH
